# Optimizing beta-blocker therapy in cardiovascular care: a national cross-sectional assessment of physicians’ and nurses’ knowledge, attitudes, and practices in Saudi Arabia

**DOI:** 10.3389/fmed.2026.1807201

**Published:** 2026-05-07

**Authors:** Firas A. Alghuthmi, Ruyuf S. Haddash, Eman O. Alsheikh, Qusai H. Sannan, Moayed M. Alharbi, Jaser F. Alharbi, Qusai H. Basranh, Ahmed A. Alsubhi, Ohood K. Almuzaini, Saad M. Wali

**Affiliations:** 1 College of Pharmacy, Umm Al-Qura University, Makkah, Saudi Arabia; 2 Department of Pharmacology and Toxicology, Faculty of Medicine, Umm Al-Qura University, Makkah, Saudi Arabia; 3 Department of Pharmacology and Toxicology, College of Pharmacy, Umm Al-Qura University, Makkah, Saudi Arabia

**Keywords:** beta-blockers, cardiovascular disease, heart failure, nurses, physicians, Saudi Arabia

## Abstract

**Introduction:**

Beta-blockers are a cornerstone of guideline directed medical therapy for cardiovascular diseases; however, their underutilization remains a global concern. Healthcare professionals’ knowledge, attitudes, and practices play a critical role in optimizing beta-blocker prescribing and monitoring. This study aimed to assess the knowledge, attitudes, and practices of physicians and nurses regarding beta-blocker use in cardiovascular patients in Saudi Arabia.

**Methods:**

A national cross-sectional study was conducted among physicians and nurses working in government hospitals across Saudi Arabia. Data were collected using a structured self-administered questionnaire assessing demographics, knowledge, attitudes and practices related to beta-blocker use and monitoring. Descriptive statistics, group comparisons, and multivariable linear regression analyses were performed.

**Results:**

A total of 553 healthcare professionals participated, including 249 physicians (45.0%) and 304 nurses (55.0%). The median knowledge score was 6.0 (IQR: 5.0–8.0). Nurses demonstrated higher knowledge in titration principles (79.3% vs. 59.8%, *p* < 0.001) and recognition of rebound effects (74.3% vs. 65.5%, *p* = 0.014). In contrast, physicians showed greater knowledge in appropriate beta-blocker selection (52.2% vs. 40.8%, *p* = 0.008) and were more likely to correctly identify that beta-blockers are not contraindicated in all patients with diabetes (42.6% vs. 31.9%, *p* = 0.017). Fear of adverse effects, particularly bradycardia and hypotension, was more frequently reported among nurses (58.6% vs. 36.1%, *p* < 0.001). Participants with 1–5 years (β = 0.61, 95% CI: 0.14–1.07, *p* = 0.010) and 6–10 years (β = 0.69, 95% CI: 0.03–1.35, *p* = 0.040) had higher knowledge scores, while frequent patient management was associated with lower knowledge (β = −0.85, 95% CI, −1.52 to −0.18, *p* = 0.013). Attitude scores were lower among nurses (β = −1.28, 95% CI, −2.08 to −0.48, *p* = 0.002) and those managing patients less frequently (β = −1.64, 95% CI, −2.99 to −0.28, *p* = 0.018).

**Conclusion:**

Healthcare professionals in Saudi Arabia demonstrated moderate knowledge regarding beta-blocker therapy, with distinct professional differences in knowledge domains and perceived barriers. Fear of adverse effects emerged as a key obstacle to optimal beta-blocker use, particularly among nurses. Targeted educational interventions, unified institutional protocols, and enhanced interprofessional collaboration are needed to improve adherence to guideline-directed beta-blocker therapy and optimize cardiovascular outcomes.

## 1 Introduction

Cardiovascular diseases (CVDs) is the leading cause of mortality worldwide, accounting for approximately 17.9 million deaths annually and representing 32% of all global deaths (1). In Saudi Arabia, CVDs are responsible for approximately 45% of all reported deaths, placing a substantial burden on the healthcare system (2). The rising prevalence of cardiovascular risk factors has contributed to the increasing incidence of CVDs in the region (3). Effective management of these conditions requires the optimization of evidence-based pharmacological therapies, among which beta-blockers play a pivotal role.

Beta-blockers represents an essential component of guideline-directed medical therapy for numerous CVDs. Both the European Society of Cardiology (ESC) and the American College of Cardiology/American Heart Association guidelines recommend beta-blockers as first-line therapy for patients with heart failure with reduced ejection fraction (HFrEF), post-myocardial infarction, stable angina, and certain arrhythmias (4, 5). Evidence-based of beta-blockers, such as bisoprolol, carvedilol, and metoprolol succinate, have been demonstrated to reduce mortality, decrease hospitalizations, improve symptoms, and promote left ventricular reverse remodeling in patients with HFrEF (6, 7). Despite this compelling evidence, beta-blockers remain significantly underutilized in clinical practice globally, with many patients either not receiving these medications or receiving suboptimal doses (8, 9).

Inadequate utilization of beta-blockers has been attributed to several factors, including concerns regarding adverse effects such as bradycardia, hypotension, and fatigue, as well as perceived contraindications in patients with comorbidities such as diabetes mellitus, chronic obstructive pulmonary disease, and peripheral arterial disease (10, 11). Healthcare providers’ knowledge, attitudes, and practices (KAP) play a pivotal role in determining prescribing patterns and adherence to clinical guidelines. Studies have demonstrated that inadequate knowledge and negative attitudes among healthcare professionals contribute significantly to the suboptimal use of evidence-based therapies (12, 13). Furthermore, proper titration of beta-blockers, following the “start low, go slow” principle, is essential for achieving therapeutic targets while minimizing adverse effects, yet this approach is often not adequately implemented in clinical settings (14).

In Saudi Arabia, limited data exist regarding healthcare providers’ understanding and application of beta-blocker therapy in cardiovascular care. Previous studies have examined pharmacists’ knowledge of cardiovascular medications and general cardiovascular disease awareness among the public (15, 16), but comprehensive assessments of physicians’ and nurses’ KAP specifically concerning beta-blockers are lacking. Given that nurses play an increasingly critical role in patient monitoring, education, and medication administration, their knowledge and attitudes are equally important in ensuring optimal therapeutic outcomes (17). Interprofessional collaboration between physicians and nurses is essential for effective cardiovascular care delivery, particularly in the initiation, titration, and monitoring of beta-blocker therapy (18).

Therefore, this study aimed to evaluate the KAP of physicians and nurses regarding the use and monitoring of beta-blockers in cardiovascular patients in government hospitals in Saudi Arabia. The specific objectives were to assess knowledge of beta-blocker indications, contraindications, and guideline-recommended uses; evaluate familiarity with titration protocols and dose-adjustment strategies; investigate monitoring practices, including recognition of adverse effects and safety parameters; explore attitudes toward beta-blocker use across different cardiovascular conditions; identify barriers to appropriate prescribing and monitoring in clinical practice; and provide recommendations for standardized protocols and interprofessional education.

## 2 Materials and methods

### 2.1 Study design

A descriptive cross-sectional study was conducted to assess the KAP of healthcare professionals regarding beta-blocker therapy in cardiovascular patients. The study employed a quantitative research design utilizing a self-administered online questionnaire distributed through electronic platforms. This design was selected because it enables efficient data collection from a geographically dispersed population and facilitates the examination of associations between professional characteristics and KAP at a single point in time.

### 2.2 Study setting and participants

The study was conducted across government hospitals in multiple regions of Saudi Arabia, including the Central, Western, Eastern, Northern, and Southern regions. There are approximately 550 government hospitals in Saudi Arabia under the Ministry of Health and other governmental sectors. Study sites were selected to ensure geographic representation across these regions.

The target population comprised licensed physicians and nurses actively practicing in clinical settings where cardiovascular patients are managed. Eligible participants included consultants, specialists, residents, general practitioners, and registered nurses with varying levels of clinical experience. Healthcare professionals were required to have direct involvement in the care of patients receiving beta-blockers for cardiovascular conditions. Participants were recruited using a convenience sampling approach based on accessibility and institutional collaboration. Although this approach may limit full national representativeness, efforts were made to include diverse regions and hospital types to enhance generalizability. Participants who were on extended leave, those working exclusively in non-clinical roles, and those who declined to provide informed consent were excluded from the study.

### 2.3 Sample size estimation

The sample size was estimated using G*Power software (version 3.1.9.7) based on comparison of the primary score outcome between physicians and nurses as two independent groups. An *a priori* analysis was conducted using the *t*-test: difference between two independent means (two-tailed). With an assumed medium effect size (Cohen’s *d* = 0.50), α = 0.05, power = 0.80, and an allocation ratio of 1:1, the minimum required sample size was 128 participants (64 per group). However, we sought to extend participants’ recruitment to include the maximum feasible number of eligible participants and enhance the robustness and generalizability of the findings.’

### 2.4 Survey instrument

A structured questionnaire was developed based on current clinical guidelines (4, 5), relevant literature (8, 11), and expert consultation. The questionnaire was pilot tested among a small group of healthcare professionals (*n* = 20) to assess clarity, comprehensiveness, and face validity. Modifications were made based on feedback received. The questionnaire demonstrated acceptable internal consistency, with Cronbach’s alpha values of 0.671 for the knowledge domain and 0.697 for the attitude domain. Content validity was ensured through expert review by clinicians with experience in cardiovascular pharmacotherapy and survey-based research.

### 2.5 Data collection

Data were collected electronically using a secure online survey platform. The questionnaire link was distributed through email invitations, professional social media groups, and hospital internal communication systems. Participation was voluntary, and responses were collected anonymously to ensure confidentiality. A reminder was sent 2 weeks after the initial invitation to maximize the response rate. Data were collected over a 3-month period.

### 2.6 Statistical analysis

Data were analyzed using RStudio (version 2024.9.1.394, Boston, MA, United States) with R version 4.4.2. Descriptive statistics were used to summarize participant characteristics, knowledge, attitudes, and practices. Categorical variables were presented as frequencies and percentages, and compared using Pearson’s chi-squared test or Fisher’s exact test where appropriate. The distribution of knowledge and attitude scores was assessed using the Shapiro–Wilk test, which indicated non-normal distribution (*p* < 0.001 for both variables); therefore, these variables were summarized using median and interquartile range (IQR) and analyzed using non-parametric tests. Continuous variables, including knowledge and attitude scores, were summarized using median and interquartile range (IQR). Group differences in these scores were assessed using the Wilcoxon rank sum test and Kruskal–Wallis rank sum test. Multivariable linear regression models were used to identify factors associated with knowledge and attitude scores, with results reported as beta coefficients and 95% confidence intervals (CI). Internal consistency of the knowledge and attitude scales was assessed using Cronbach’s alpha. A two-tailed *p*-value of less than 0.05 was considered statistically significant.

## 3 Results

### 3.1 Demographic and occupational characteristics of the participants

**FIGURE 1:**
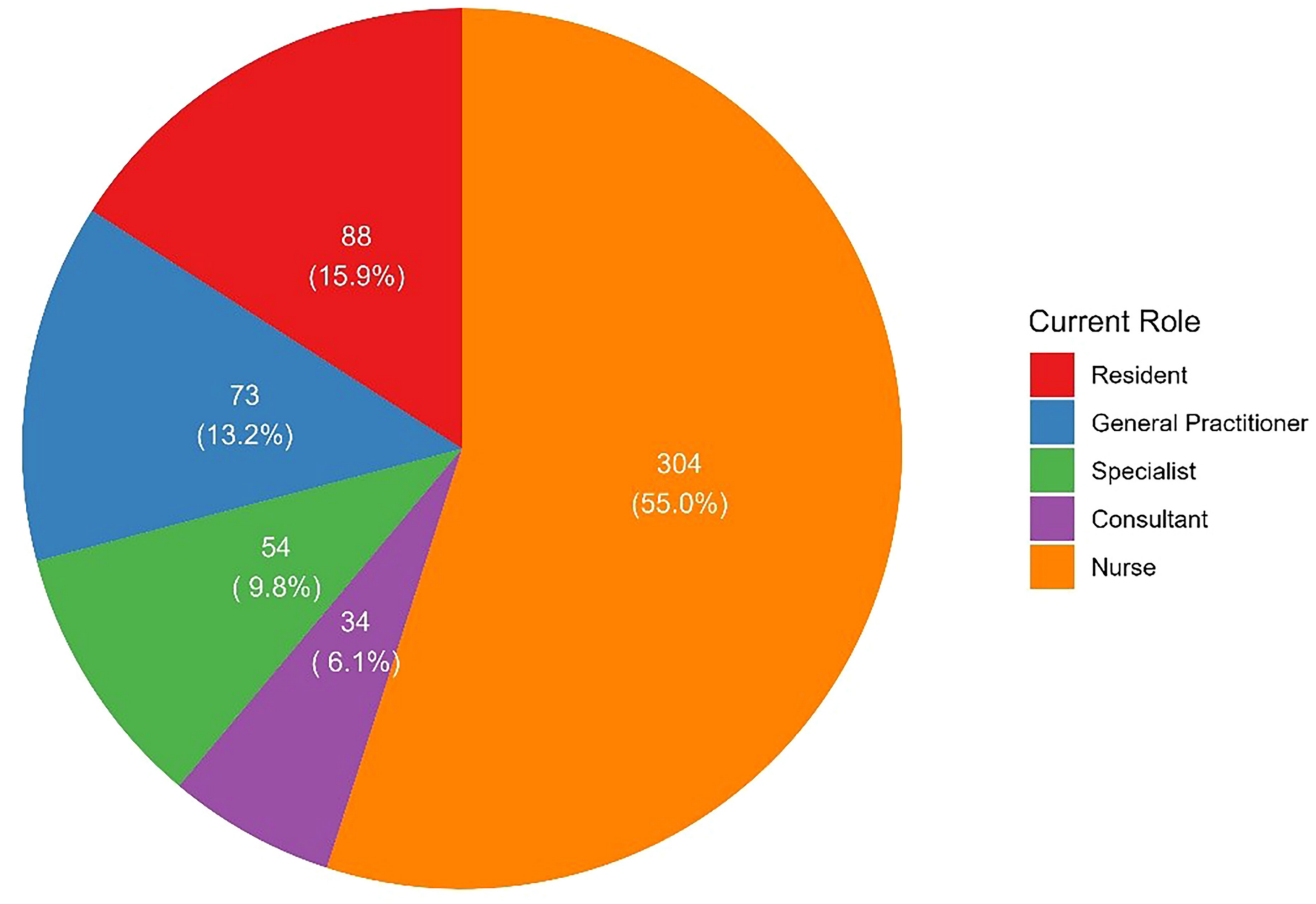
Frequencies and proportions of participants’ roles.

Among the 553 participants, the gender distribution was nearly equal, with 49.5% males and 50.5% females. The majority of participants were aged 25–34 years (75.0%), followed by 18.6% aged 35–44 years. Most respondents were Saudi nationals (69.8%). In terms of professional role, nurses comprised more than a half of the sample (55.0%, *n* = 304), followed by residents (15.9%, *n* = 88), general practitioners (13.2%, *n* = 73), specialists (9.8%, *n* = 54), and consultants (6.1%, *n* = 34, Figure 1). Regarding years of practice, 42.5% had 1–5 years of experience. The frequency of managing patients receiving beta-blockers varied, with 37.8% reporting doing so several times a week, 32.9% daily, 18.1% occasionally, and 11.2% rarely (Table 1).

**TABLE 1:** Demographic and occupational characteristics of the participants.

Characteristic	Overall *N* = 553	Physician *N* = 249	Nurse *N* = 304	*p*-value
Gender				< 0.001
Male	274 (49.5%)	182 (73.1%)	92 (30.3%)	
Female	279 (50.5%)	67 (26.9%)	212 (69.7%)	
Age				0.008
25–34	415 (75.0%)	175 (70.3%)	240 (78.9%)	
35–44	103 (18.6%)	49 (19.7%)	54 (17.8%)	
45–54	26 (4.7%)	18 (7.2%)	8 (2.6%)	
55–64	9 (1.6%)	7 (2.8%)	2 (0.7%)	
Nationality				< 0.001
Saudi	386 (69.8%)	201 (80.7%)	185 (60.9%)	
Non-Saudi	167 (30.2%)	48 (19.3%)	119 (39.1%)	
Years in practice				0.040
Less than 1 year	153 (27.7%)	57 (22.9%)	96 (31.6%)	
1–5 years	235 (42.5%)	105 (42.2%)	130 (42.8%)	
6–10 years	90 (16.3%)	45 (18.1%)	45 (14.8%)	
More than 10 years	75 (13.6%)	42 (16.9%)	33 (10.9%)	
Frequency of managing patients who receive beta-blockers for cardiovascular conditions				< 0.001
Rarely	62 (11.2%)	0 (0.0%)	62 (20.4%)	
Occasionally	100 (18.1%)	50 (20.1%)	50 (16.4%)	
Several times a week	209 (37.8%)	117 (47.0%)	92 (30.3%)	
Daily	182 (32.9%)	82 (32.9%)	100 (32.9%)	

n (%). Pearson’s Chi-squared test; Fisher’s exact test.

Significant differences were observed between physicians and nurses across several demographic and occupational characteristics. Male participants predominated among physicians (73.1%) while the majority of nurses were female (69.7%, *p* < 0.001). Nurses were younger, with 78.9% aged 25–34 years compared to 70.3% of physicians (*p* = 0.008). A greater proportion of physicians were Saudi nationals (80.7% vs. 60.9%, *p* < 0.001). Nurses were more likely to have less than 1 year of experience (31.6% vs. 22.9%, *p* = 0.040). Nurses were more likely to report rarely managing patients on beta-blockers (20.4%), whereas none of the physicians selected this response. Conversely, a higher proportion of physicians reported managing such patients several times a week (47.0% vs. 30.3%, *p* < 0.001, Table 1).

### 3.2 Knowledge of beta-blocker use and monitoring

Nurses demonstrated significantly higher knowledge than physicians in several key areas related to beta-blocker use and monitoring. A greater proportion of nurses correctly identified that beta-blockers are guideline-recommended for patients with HFrEF (78.9% vs. 75.5%, *p* = 0.002) and that their titration in heart failure should follow the “start low, go slow” principle (79.3% vs. 59.8%, *p* < 0.001). Additionally, more nurses recognized that sudden discontinuation of beta-blockers may result in rebound hypertension or angina (74.3% vs. 65.5%, *p* = 0.014). Nurses were also more likely than physicians to correctly report that beta-blockers can be used safely in most hypertensive patients (67.8% vs. 50.6%, *p* < 0.001, Table 2).

**TABLE 2:** Participants’ responses to knowledge items.

Characteristic	Physician *N* = 249	Nurse *N* = 304	*p*-value
Beta-blockers are guideline-recommended for patients with reduced ejection fraction heart failure			0.002
True*	188 (75.5%)	240 (78.9%)	
False	34 (13.7%)	17 (5.6%)	
Do not know	27 (10.8%)	47 (15.5%)	
Beta-blockers improve mortality and morbidity in ischemic heart disease			0.021
True*	159 (63.9%)	159 (52.3%)	
False	16 (6.4%)	30 (9.9%)	
Do not know	74 (29.7%)	115 (37.8%)	
Any beta-blocker (including atenolol and propranolol) is appropriate for heart failure with reduced ejection fraction			0.008
True	62 (24.9%)	77 (25.3%)	
False*	130 (52.2%)	124 (40.8%)	
Do not know	57 (22.9%)	103 (33.9%)	
Beta-blockers should always be avoided in patients with asthma			0.010
True	117 (47.0%)	120 (39.5%)	
False*	105 (42.2%)	123 (40.5%)	
Do not know	27 (10.8%)	61 (20.1%)	
Monitoring heart rate and blood pressure is essential after beta-blocker initiation			0.882
True*	183 (73.5%)	223 (73.4%)	
False	17 (6.8%)	18 (5.9%)	
Do not know	49 (19.7%)	63 (20.7%)	
Titration of beta-blockers in heart failure should follow the “start low, go slow” principle			< 0.001
True*	149 (59.8%)	241 (79.3%)	
False	15 (6.0%)	32 (10.5%)	
Do not know	85 (34.1%)	31 (10.2%)	
Sudden discontinuation of beta-blockers may lead to rebound hypertension or angina			0.014
True*	163 (65.5%)	226 (74.3%)	
False	16 (6.4%)	24 (7.9%)	
Do not know	70 (28.1%)	54 (17.8%)	
Patient education is essential for ensuring beta-blocker adherence			0.014
True*	223 (89.6%)	248 (81.6%)	
False	12 (4.8%)	17 (5.6%)	
Do not know	14 (5.6%)	39 (12.8%)	
Beta-blockers can be used safely in most hypertensive patients			< 0.001
True*	126 (50.6%)	206 (67.8%)	
False	81 (32.5%)	40 (13.2%)	
Do not know	42 (16.9%)	58 (19.1%)	
Beta-blockers are contraindicated in all patients with diabetes			0.017
True	104 (41.8%)	138 (45.4%)	
False*	106 (42.6%)	97 (31.9%)	
Do not know	39 (15.7%)	69 (22.7%)	

*An asterisk indicates a correct response. n (%). Pearson’s Chi-squared test.

On the other hand, a greater proportion of physicians correctly identified that beta-blockers improve mortality and morbidity in ischemic heart disease (63.9% vs. 52.3%, *p* = 0.021). Physicians were also more likely to correctly reject the statement that any beta-blocker, including atenolol and propranolol, is appropriate for HFrEF (52.2% vs. 40.8%, *p* = 0.008). In addition, physicians more frequently recognized that beta-blockers should not always be avoided in patients with asthma (42.2% vs. 40.5%, *p* = 0.010) and that patient education is essential for ensuring adherence to beta-blocker therapy (89.6% vs. 81.6%, *p* = 0.014). Furthermore, a higher proportion of physicians correctly identified that beta-blockers are not contraindicated in all patients with diabetes compared with nurses (42.6% vs. 31.9%, *p* = 0.017, Table 2).

### 3.3 Factors associated with knowledge scores

In general, the median knowledge score among participants was 6.0, with an interquartile range (IQR) of 5.0–8.0. Internal consistency, assessed using Cronbach’s alpha, was acceptable, with α = 0.671 for the 10-item knowledge scale.

**FIGURE 2:**
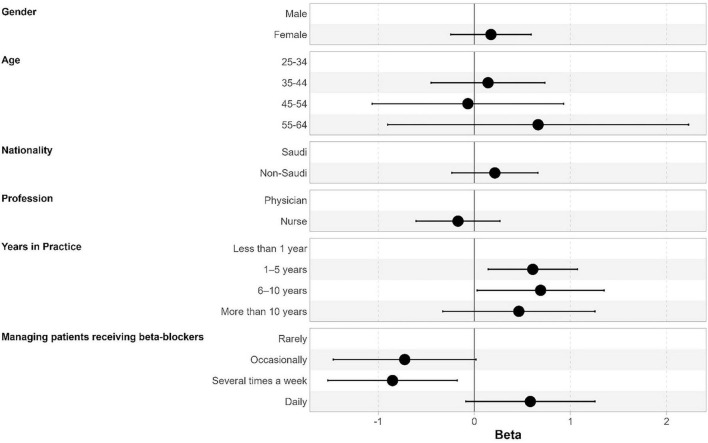
Factors associated with knowledge scores. Results are expressed as beta coefficients, and error bars represent 95% confidence intervals.

Participants with 1–5 years (beta = 0.61, 95% CI, 0.14–1.07, *p* = 0.010) and 6–10 years of experience (beta = 0.69, 95% CI, 0.03–1.35, *p* = 0.040) had significantly higher knowledge scores compared to those with less than 1 year of experience. Those managing beta-blocker patients several times a week had lower knowledge scores than those managing them rarely (beta = −0.85, 95% CI, −1.52 to −0.18, *p* = 0.013). Profession (physicians vs. nurses) was not an independent predictor of knowledge (Table 3 and Figure 2).

**TABLE 3:** Factors associated with knowledge of beta-blockers use and monitoring in patients with cardiovascular diseases.

Variable	Knowledge score		Multivariable regression		
	Median (IQR)	*p*-value	Beta	95% CI	*p*-value
Gender		0.120			
Male	6.0 (5.0, 8.0)		Reference	Reference	
Female	7.0 (5.0, 8.0)		0.17	−0.25, 0.59	0.417
Age		0.169			
25–34	6.0 (5.0, 8.0)		Reference	Reference	
35–44	7.0 (5.0, 8.0)		0.14	−0.45, 0.73	0.635
45–54	6.0 (5.0, 8.0)		−0.07	−1.06, 0.93	0.896
55–64	7.0 (6.0, 8.0)		0.66	−0.90, 2.23	0.405
Nationality		0.006			
Saudi	6.0 (4.0, 8.0)		Reference	Reference	
Non-Saudi	7.0 (5.0, 8.0)		0.21	−0.23, 0.66	0.350
Profession		0.433			
Physician	6.0 (5.0, 8.0)		Reference	Reference	
Nurse	7.0 (5.0, 8.0)		−0.17	−0.61, 0.27	0.447
Years in practice		0.003			
Less than 1 year	6.0 (4.0, 8.0)		Reference	Reference	
1–5 years	7.0 (5.0, 8.0)		0.61	0.14, 1.07	0.010
6–10 years	7.0 (6.0, 8.0)		0.69	0.03, 1.35	0.040
More than 10 years	6.0 (5.0, 9.0)		0.46	−0.33, 1.25	0.251
Frequency of managing patients who receive beta-blockers for cardiovascular conditions		< 0.001			
Rarely	8.0 (5.0, 8.0)		Reference	Reference	
Occasionally	6.0 (5.0, 7.0)		−0.72	−1.47, 0.02	0.056
Several times a week	5.0 (4.0, 7.0)		−0.85	−1.52, −0.18	0.013
Daily	7.0 (6.0, 9.0)		0.58	−0.09, 1.25	0.089

IQR, interquartile range. Wilcoxon rank sum test; Kruskal-Wallis rank sum test. CI, confidence interval.

### 3.4 Attitudes toward beta-blocker use and monitoring

Overall, 62.7% of physicians and 55.9% of nurses expressing confidence in prescribing and titrating beta-blockers (*p* = 0.048). A higher proportion of nurses agreed that beta-blockers remain underutilized (53.0% vs. 36.9%, *p* < 0.001) and that patients frequently discontinue due to side effects (63.5% vs. 45.4%, *p* < 0.001). Nurses were also more likely to agree that they avoid beta-blockers due to fear of bradycardia or hypotension (58.6% vs. 36.1%, *p* < 0.001). Agreement on the critical role of nurses in monitoring was high in both groups, but more pronounced among nurses (81.6% vs. 71.1%, *p* = 0.006, Table 4).

**TABLE 4:** Participants’ responses to attitudes items.

Characteristic	Physician *N* = 249	Nurse *N* = 304	*p*-value
I feel confident in prescribing and titrating beta-blockers			0.048
Agree/Strongly agree	156 (62.7%)	170 (55.9%)	
Neutral	75 (30.1%)	120 (39.5%)	
Strongly disagree/disagree	18 (7.2%)	14 (4.6%)	
Beta-blockers are underused in cardiovascular practice in my hospital.			< 0.001
Agree/Strongly agree	92 (36.9%)	161 (53.0%)	
Neutral	74 (29.7%)	69 (22.7%)	
Strongly disagree/disagree	83 (33.3%)	74 (24.3%)	
Nurses play a critical role in monitoring patients on beta-blockers.			0.006
Agree/Strongly agree	177 (71.1%)	248 (81.6%)	
Neutral	63 (25.3%)	44 (14.5%)	
Strongly disagree/disagree	9 (3.6%)	12 (3.9%)	
I believe there are adequate institutional protocols for beta-blocker use			0.526
Agree/Strongly agree	167 (67.1%)	217 (71.4%)	
Neutral	61 (24.5%)	63 (20.7%)	
Strongly disagree/disagree	21 (8.4%)	24 (7.9%)	
Patients frequently discontinue beta-blockers due to side effects			< 0.001
Agree/Strongly agree	113 (45.4%)	193 (63.5%)	
Neutral	81 (32.5%)	75 (24.7%)	
Strongly disagree/disagree	55 (22.1%)	36 (11.8%)	
I often avoid beta-blockers due to fear of bradycardia or hypotension			< 0.001
Agree/Strongly agree	90 (36.1%)	178 (58.6%)	
Neutral	70 (28.1%)	84 (27.6%)	
Strongly disagree/disagree	89 (35.7%)	42 (13.8%)	
I refer to ESC or AHA guidelines when prescribing beta-blockers			0.289
Agree/strongly agree	181 (72.7%)	203 (66.8%)	
Neutral	54 (21.7%)	77 (25.3%)	
Strongly disagree/disagree	14 (5.6%)	24 (7.9%)	
Interprofessional collaboration (physicians, nurses, pharmacists) is needed to optimize beta-blocker use			0.600
Agree/strongly agree	195 (78.3%)	233 (76.6%)	
Neutral	44 (17.7%)	62 (20.4%)	
Strongly disagree/disagree	10 (4.0%)	9 (3.0%)	
Training workshops on cardiovascular pharmacotherapy should be regularly provided.			0.098
Agree/strongly agree	204 (81.9%)	228 (75.0%)	
Neutral	37 (14.9%)	57 (18.8%)	
Strongly disagree/disagree	8 (3.2%)	19 (6.3%)	
Monitoring parameters (e.g., ECG, pulse, BP) are always followed in my clinical setting			0.834
Agree/strongly agree	188 (75.5%)	236 (77.6%)	
Neutral	49 (19.7%)	54 (17.8%)	
Strongly disagree/disagree	12 (4.8%)	14 (4.6%)	

n (%). Pearson’s Chi-squared test.

### 3.5 Factors associated with attitude scores

**FIGURE 3:**
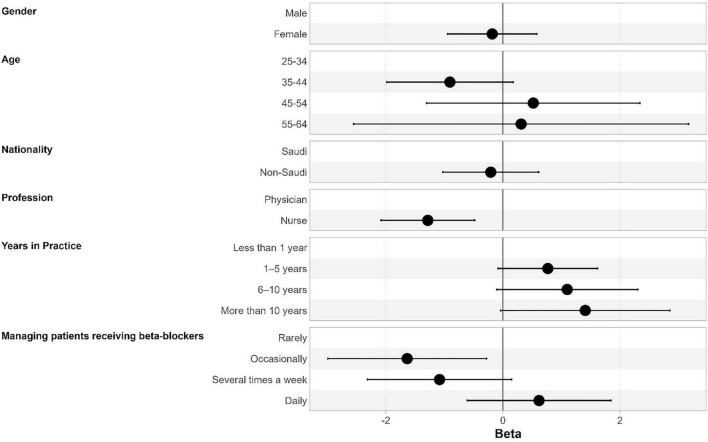
Factors associated with attitudes scores. Results are expressed as beta coefficients, and error bars represent 95% confidence intervals.

For attitudes, the median score was 36.0, with an IQR of 34.0–39.0. Regarding the internal consistency, the Cronbach’s α was 0.697 for the 10-item attitude scale. Attitude scores were significantly associated with profession and frequency of managing patients on beta-blockers. Nurses had lower attitude scores than physicians (beta = −1.28, 95% CI, −2.08 to −0.48, *p* = 0.002). Those managing patients occasionally had lower scores than those managing them rarely (beta = −1.64, 95% CI, −2.99 to −0.28, *p* = 0.018, Table 5 and Figure 3).

**TABLE 5:** Factors associated with positive attitudes toward beta-blockers use and monitoring in patients with cardiovascular diseases.

Variable	Attitudes score		Multivariable regression		
	Median (IQR)	*p*-value	Beta	95% CI	*p*-value
Gender		0.173			
Male	36.5 (34.0, 40.0) 36.0 (34.0, 38.0)		Reference	Reference	0.640
Female			−0.18	−0.95, 0.58	
Age		0.262			
25–34	36.0 (34.0, 39.0) 36.0 (33.0, 39.0)		Reference	Reference	0.101
35–44			−0.91	−1.99, 0.18	
45–54	37.5 (35.0, 40.0)		0.52	−1.30, 2.34	0.576
55–64	37.0 (35.0, 39.0)		0.31	−2.55, 3.17	0.831
Nationality		0.924			
Saudi	36.0 (34.0, 39.0) 36.0 (34.0, 39.0)		Reference	Reference	0.620
Non-Saudi			−0.21	−1.03, 0.61	
Profession		< 0.001			
Physician	37.0 (34.0, 40.0) 36.0 (34.0, 38.0)		Reference	Reference	0.002
Nurse			−1.28	−2.08, −00.48	
Years in Practice		0.092			0.076
Less than 1 year	36.0 (33.0, 38.0) 36.0 (34.0, 39.0)		Reference	Reference	
1–5 years			0.77	−0.08, 1.62	
6–10 years	37.0 (34.0, 40.0)		1.10	−.11, 2.31	0.074
More than 10 years	36.0 (34.0, 40.0)		1.41	−0.04, 2.86	0.056
Frequency of managing patients who receive beta-blockers for cardiovascular conditions		< 0.001			
Rarely	36.0 (35.0, 38.0) 35.0 (33.0, 38.0)		Reference	Reference	0.018
Occasionally			−1.64	−2.99, −0.28	
Several times a week	36.0 (33.0, 38.0)		−1.08	−2.31, 0.15	0.085
Daily	37.0 (35.0, 41.0)		0.62	−0.61, 1.85	0.323

IQR’, interquartile range. Wilcoxon rank sum test; Kruskal-Wallis rank sum test. CI, Confidence Interval.

### 3.6 Practices related to beta-blocker use

Heart failure was the most commonly reported condition for beta-blocker use among both physicians (75.9%) and nurses (76.0%). A significant proportion of physicians reported their use in post-myocardial infarction (45.0% vs. 29.6%, *p* < 0.001). Most participants reported titrating beta-blockers by starting with a low dose and gradually increasing it (64.3% of physicians vs. 70.4% of nurses, *p* = 0.049). Nurses were more likely to agree that beta-blockers are discontinued due to side effects (67.8% vs. 55.4%, *p* = 0.003). A statistically significant difference was observed in monitoring practices during beta-blocker therapy, with 45.8% of physicians and 53.6% of nurses reporting regular heart rate and blood pressure monitoring during initiation or titration. Among all essential parameters for patient monitoring, documentation of heart rate was the most commonly reported in practice, particularly among nurses compared to physicians. (63.8% vs. 53.8%, *p* = 0.004). Fear of side effects was the most frequently reported barrier to optimal use of beta-blocker in nurses compared to physician (69.4% vs. 57.8%, *p* = 0.005) (Table 6).

**TABLE 6:** Participants’ responses to practice items.

Characteristic	Physician *N* = 249	Nurse *N* = 304	*p*-value
Conditions where beta-blockers are commonly prescribed/administered*			
Angina	104 (41.8%)	137 (45.1%)	0.436
Arrhythmia	104 (41.8%)	145 (47.7%)	0.163
Atrial fibrillation	122 (49.0%)	154 (50.7%)	0.697
Post-myocardial infarction	112 (45.0%)	90 (29.6%)	< 0.001
Heart failure	189 (75.9%)	231 (76.0%)	0.982
Hypertension	172 (69.1%)	219 (72.0%)	0.446
Typical titration practice for beta-blockers in heart failure patients			0.049
Let specialist handle titration	48 (19.3%)	34 (11.2%)	
Start at target dose	29 (11.6%)	44 (14.5%)	
Start with a low dose and titrate gradually	160 (64.3%)	214 (70.4%)	
Not applicable	12 (4.8%)	12 (3.9%)	
Ever discontinued a beta-blocker due to side effects	138 (55.4%)	206 (67.8%)	0.003
Side effects most commonly monitored			0.122
Hypotension	47 (18.9%)	62 (20.4%)	
Bradycardia	151 (60.6%)	185 (60.9%)	
Fatigue	25 (10.0%)	35 (11.5%)	
Wheezing/asthma exacerbation	22 (8.8%)	12 (3.9%)	
Cold extremities	4 (1.6%)	10 (3.3%)	
Frequency of monitoring heart rate and BP during beta-blocker initiation or titration			0.080
Never	33 (13.3%)	44 (14.5%)	
Rarely	7 (2.8%)	12 (3.9%)	
Sometimes	38 (15.3%)	42 (13.8%)	
Often	57 (22.9%)	43 (14.1%)	
Always	114 (45.8%)	163 (53.6%)	
Parameters documented when monitoring beta-blocker use			0.004
ECG	24 (9.6%)	31 (10.2%)	
Heart rate	134 (53.8%)	194 (63.8%)	
Blood pressure	54 (21.7%)	63 (20.7%)	
Patient-reported side effects	28 (11.2%)	12 (3.9%)	
None	9 (3.6%)	4 (1.3%)	
Informing patients about potential side effects and importance of adherence	219 (88.0%)	258 (84.9%)	0.295
Main barriers to optimal beta-blocker use*			
Fear of side effects	144 (57.8%)	211 (69.4%)	0.005
Lack of protocols	71 (28.5%)	87 (28.6%)	0.978
Patient non-adherence	146 (58.6%)	171 (56.3%)	0.573
Time constraints	102 (41.0%)	116 (38.2%)	0.502

*An asterisk indicates a multiple-response item. n (%). Pearson’s Chi-squared test.

## 4 Discussion

This national cross-sectional study assessed the knowledge, attitudes, and practices (KAP) of physicians and nurses regarding beta-blocker therapy in cardiovascular care in Saudi Arabia. A significant professional difference in practical knowledge, prescribing confidence, and perceived limitations to optimal beta-blocker use were identified, along with relatively moderate levels of expertise. Interestingly, nurses demonstrated superior knowledge in areas related to practical application and patient safety, while physicians exhibited greater awareness of evidence-based indications and drug selection. However, the fear of adverse effects remained a significant barrier to optimal beta-blocker utilization among nurses. These findings have a critical implication for clinical education and practice improvement initiatives in academia and clinical practice which highlight the need to the appropriate use of beta-blocker.

### 4.1 Fear of adverse effects as a key barrier to beta-blocker use

The knowledge assessment revealed several strengths and gaps among healthcare professionals. Pervious findings showed nurses have higher awareness of titration principles and rebound effects is consistent with their direct involvement in medication administration and patient monitoring (19). Nurses are often responsible for recognizing early signs of adverse effects and ensuring gradual dose adjustments, which may explain their greater familiarity with these practical aspects (17). Conversely, physicians demonstrated a greater understanding on evidence-based drug selection, recognizing that not all beta-blockers are appropriate for heart failure, which aligns with their prescribing responsibilities (20). Such variations in knowledge underscores the importance of interprofessional collaboration, whereby the distinct expertise of each professional group contributes to comprehensive patient care (18).

Interestingly, in assessment of their attitude a significant proportion of nurses (58.6%) reported avoiding beta-blockers due to fear of bradycardia or hypotension is concerning, such factor may contribute to the widely recognized inadequate use of these life-saving medications (8, 21). This finding is consistent with international literature suggesting that concerns regarding hemodynamics effects represent a major barrier to beta-blocker optimizations, despite evidence that these medications are well-tolerated when properly titrated (10, 22). Importantly, the recent reported guidelines of ESC (2023) emphasize that beta-blockers remain one of the “foundational four” therapies for HFrEF and should be initiated and up titrated in all eligible patients (5). Thus, educational interventions addressing the misconceptions about adverse effects and emphasizing the safety of gradual titration may help overcome these attitudinal barriers.

The practice patterns observed in this study largely aligned with current guideline recommendations. The majority of participants correctly identified heart failure as a primary indication for beta-blocker therapy and reported following the “start low, go slow” titration approach. However, the finding that fear of side effects was the most reported barrier to optimal use suggests a disconnect between knowledge and practice. Studies have demonstrated that suboptimal dosing of beta-blockers is associated with poorer clinical outcomes, including increased mortality and hospitalization rates (23, 24). The STRONG-HF trial demonstrated that rapid uptitration of guideline-directed therapies, including beta-blockers, within 6 weeks of discharge significantly reduced heart failure hospitalizations and mortality (25). These findings emphasize the need for systematic protocols that support healthcare providers in overcoming barriers to optimal medication titration.

Our findings are consistent with studies from other settings examining healthcare providers’ KAP regarding cardiovascular medications. A multi-center study conducted in Saudi Arabia found that 75.5% of nurses demonstrated poor knowledge of high-risk cardiovascular medications, highlighting the need for enhanced pharmacological education (26). Similarly, studies examining pharmacists’ knowledge of cardiovascular disease prevention in Saudi Arabia identified significant gaps in understanding drug interactions and resistant hypertension management (15). These collective findings suggest that knowledge deficits regarding cardiovascular pharmacotherapy are widespread among healthcare professionals in the region and require comprehensive educational interventions.

The regression analyses identified years of experience as a significant predictor of knowledge, with those having 1–10 years of experience demonstrating higher scores than those with less than 1 year. This finding suggests that the development of beneficial information to the acquisition of practical knowledge regarding beta-blocker medication increases with clinical exposure. Noteworthy, the present study showed that those managing patients several times a week had lower knowledge scores than those managing them rarely, which may reflect the heterogeneity of the “several times a week” group or indicate that frequency of exposure alone, without structured education, does not guarantee knowledge improvement (27). The lack of association between profession and knowledge scores in the multivariable analysis suggests that differences observed in bivariate comparisons are largely explained by other factors such as experience and practice patterns.

### 4.2 Limitations and future directions

Several limitations should be considered when interpreting these findings. First, the cross-sectional design prevents establishing causal relationships between variables. Second, the use of convenience sampling may limit the generalizability of results to the broader population of healthcare professionals in Saudi Arabia. Third, self-reported data may be subject to social desirability bias, and actual clinical practices may differ from those reported. Fourth, the questionnaire assessed theoretical knowledge, which may not directly translate to clinical performance. Fifth, the internal consistency of the knowledge scale, while acceptable (α = 0.671), suggests some heterogeneity in the assessed items. Future studies should employ longitudinal designs to examine changes in KAP following educational interventions, utilize objective measures of clinical practice such as chart reviews, and include other healthcare professionals such as pharmacists who play an important role in medication management. Development of standardized competency assessments and interprofessional educational programs targeting beta-blocker optimization would be valuable contributions to improve patient outcomes. Additionally, the young age of participants and the predominance of early-career professionals may limit the generalizability of findings to more senior clinicians.

## 5 Conclusion

This study showed physicians and nurses in Saudi Arabia have a considerable experience and knowledge regarding beta-blocker therapy in cardiovascular care, with distinct areas of strength and weakness between professional groups. Fear of adverse effects, particularly bradycardia and hypotension, observed as a significant barrier to optimal beta-blocker utilization, especially among nurses. These findings highlight the need for targeted educational interventions to enhance perceptions, address knowledge gaps, implement standardized protocols, and utilize the multidisciplinary expertise of physicians and nurses to improve patient outcomes.

## Data Availability

The raw data supporting the conclusions of this article will be made available by the authors, without undue reservation.
